# Correction: Fgf-signaling is compartmentalized within the mesenchyme and controls proliferation during salamander limb development

**DOI:** 10.7554/eLife.72022

**Published:** 2021-07-09

**Authors:** Sruthi Purushothaman, Ahmed Elewa, Ashley W Seifert

Purushothaman S, Elewa A, Seifert AW. 2019. Fgf-signaling is compartmentalized within the mesenchyme and controls proliferation during salamander limb development. *eLife* **8**:e48507. doi: 10.7554/eLife.48507.

Published 20, September 2019

In the original version of our manuscript, we used a previously published scRNA-seq dataset (Gerber et al., 2018; PMID: 30262634, doi:10.1126/science.aaq0681). After contacting the authors of the published dataset to clarify an ambiguity in their Supplementary Materials, we discovered that we treated the counts in their scRNA-seq dataset as raw counts when in fact they were already normalized and logged (log2(TPM)). Thus, the analysis we published used values that were normalized twice. After re-analysing the data and comparing the outcome to our original data we found that it did not affect any of our conclusions. However, the re-analysed data does slightly affect the visual representation of data presented in Figure 4 and in the two associated Suppl. figures.

The corrected Figure 4 is shown here:

**Figure 1:**
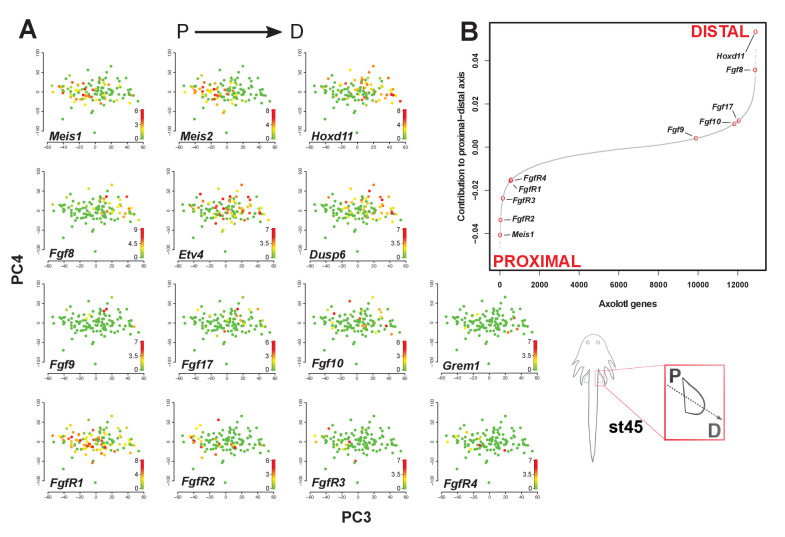


The originally published Figure 4 is shown here for reference:

**Figure 2:**
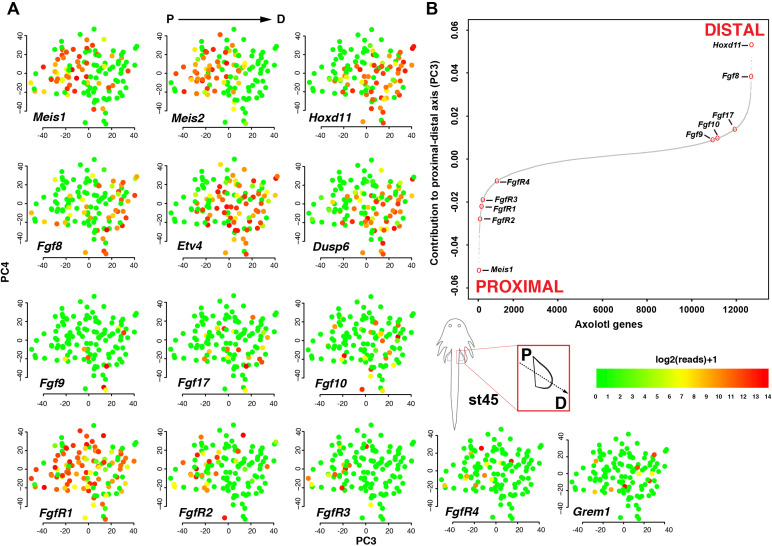


The original figure legend for Figure 4 has changed slightly (changes are underlined).

The sigmoidal curve follows a normal distribution (Shapiro-Wilks normality test, *W* = 0.95551, p<2.2e-16).

To

The sigmoidal curve follows a normal distribution (Shapiro-Wilks normality test, W = 0.96251, p<2.2e-16). Color scale legends reflect log2(TPM) values.

The corrected Figure 4 – figure supplement 1 is shown here:

**Figure 3:**
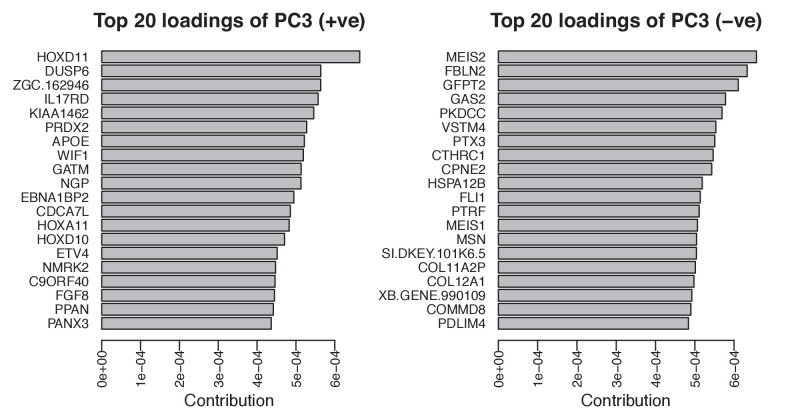


The originally published Figure 4 – figure supplement 1 is shown here for reference:

**Figure 4:**
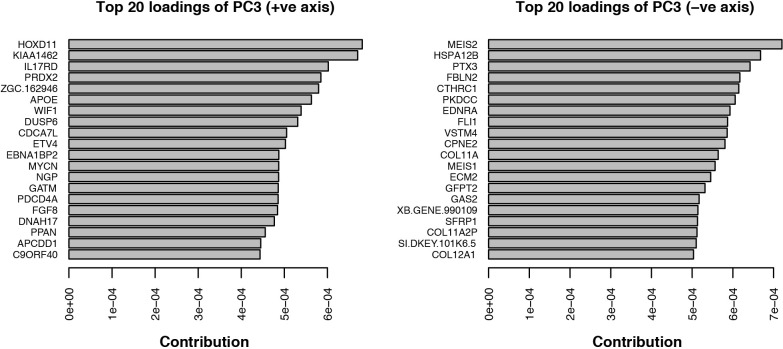


The corrected Figure 4 – figure supplement 2 is shown here:

**Figure 5:**
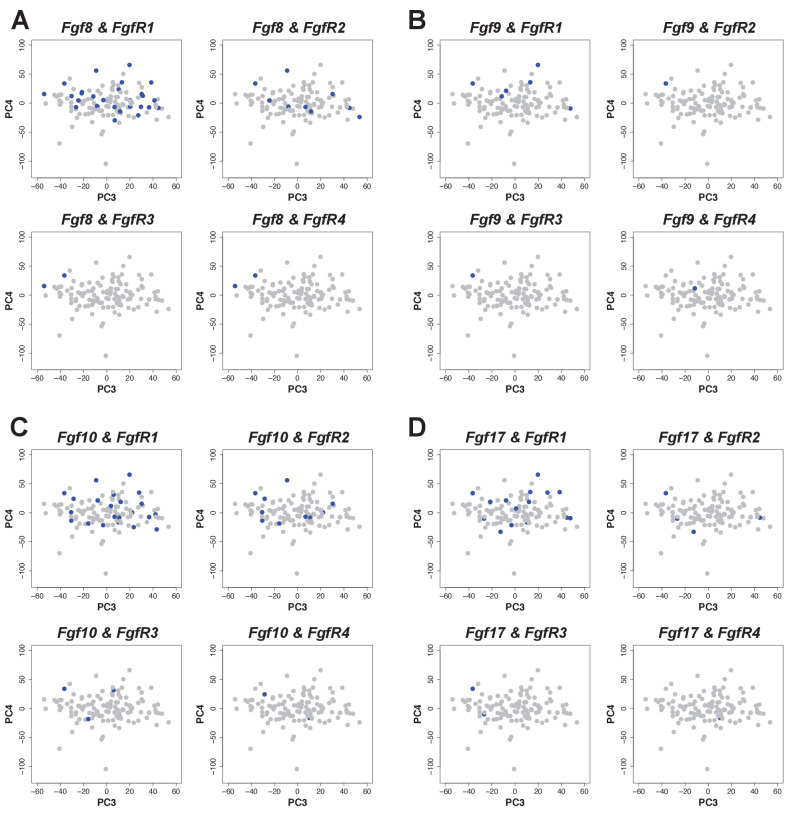


The originally published Figure 4 – figure supplement 2 is shown here for reference:

**Figure 6:**
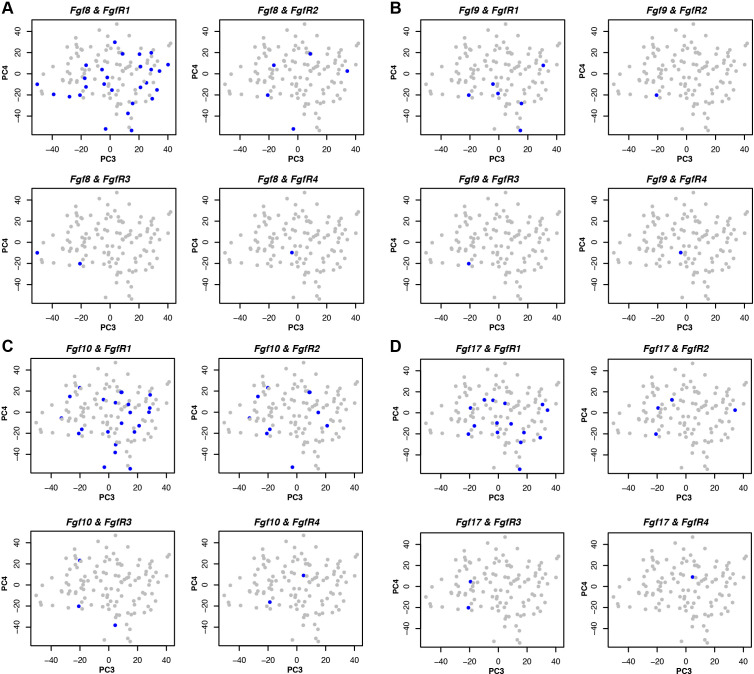


The original figure legend for Figure 4 – figure supplement 2 has changed slightly (changes are underlined).

Expression was defined as one or more RNA-seq reads mapped to the gene in question.

To:

Expression was defined as one or more transcripts per million.

In addition to the figures, a *T* and p statistic in the Main text for the Welch Two Sample t-test changed from: *T* = −4.1588, p = 0.0038, TO: *T* = -3.8104, p = 0.0064

Lastly, we have updated our methods section to reflect the single normalization.

Single-cell RNA-seq data from embryonic limb buds (Gerber et al., 2018, Table S7) were analyzed as follows: read counts from stages 40 and 44 (as reported in Gerber), and which correspond to our stages 44 and 45 (Prayag Murawala, personal communication) were normalized and used as input for principle component analysis. The top twenty loadings of the positive and negative axes of the first five principle components were inspected to identify principle components that segregated developmental axes markers on separate ends. No reliable anterior-posterior, or dorsal-ventral markers segregated in this manner in the current dataset. However, proximal-distal markers (*Meis2* and *Hoxd11*) were segregated on opposite ends of principle component 3 in the PCA of cells from stage 44, but not 40. Due to the low expression levels of most genes of interest, raw read counts were used for visualizing gene expression levels on PCA plots by rescaling the log2 of the read count to span between 0 and 14. To determine co-expression of *Fgf* ligands and receptors, expression was defined as the presence of one or more reads mapping to the gene in question.

To:

Analysis of single-cell RNA-seq data Single-cell RNA-seq data from embryonic limb buds (Gerber et al., 2018, Table S7) were analyzed as follows: Gene expression (log2(TPM)) from stages 40 and 44 (as reported in Gerber), and which correspond to our stages 44 and 45 (Prayag Murawala, personal communication) were used as input for principal component analysis. The top twenty loadings of the positive and negative axes of the first five principal components were inspected to identify principal components that segregated developmental axes markers on separate ends. No reliable anterior-posterior, or dorsal-ventral markers segregated in this manner in the current dataset. However, proximal-distal markers (Meis2 and Hoxd11) were segregated on opposite ends of principle component three in the PCA of cells from stage 44, but not 40. In order to orient PC3 so that Hoxd11 expression is on the right and Meis2 on the left (i.e., conventional proximal-distal orientation), PC3 values were multiplied by -1. To determine co-expression of *Fgf* ligands and receptors, expression was defined as the presence of one or more transcript per million.

Also, the methods listed the incorrect concentration of EdU.

Larvae were additionally treated with 0.1 ug/ml of EdU at stage 46 for 24 hr

To:

Larvae were additionally treated with 0.1 mg/ml of EdU at stage 46 for 24 hr

The article has been corrected accordingly.

